# BriGHT: transcriptome-regularized multimodal neuroimaging for brain disorder prediction

**DOI:** 10.1093/bioinformatics/btag681

**Published:** 2026-09-11

**Authors:** Zhoujie Fan, Haoran Luo, Huilong Zhou, Hao Meng, Hong Liang, Zheng Wang, Huazhu Fu, Ching-Yu Cheng, Shan Cong, Xiaohui Yao

**Affiliations:** College of Intelligent Systems Science and Engineering, Harbin Engineering University, Harbin, 150001, China; Qingdao Innovation and Development Center, Harbin Engineering University, Qingdao, 266000, China; Sanya Nanhai Innovation and Development Base, Harbin Engineering University, Sanya, 572024, China; College of Intelligent Systems Science and Engineering, Harbin Engineering University, Harbin, 150001, China; School of Hotel and Tourism Management, Hong Kong Polytechnic University, Hong Kong, 999077, China; College of Intelligent Systems Science and Engineering, Harbin Engineering University, Harbin, 150001, China; Qingdao Innovation and Development Center, Harbin Engineering University, Qingdao, 266000, China; College of Intelligent Systems Science and Engineering, Harbin Engineering University, Harbin, 150001, China; College of Intelligent Systems Science and Engineering, Harbin Engineering University, Harbin, 150001, China; Sanya Nanhai Innovation and Development Base, Harbin Engineering University, Sanya, 572024, China; School of Psychological and Cognitive Sciences, Peking University, Beijing, 100871, China; School of Biomedical Engineering, Hainan University, Sanya, 572025, China; Institute of Advanced Intelligence and Computing (IAIC), Agency for Science, Technology and Research, Singapore, 138632, Singapore; Department of Ophthalmology, Yong Loo Lin School of Medicine, National University of Singapore, Singapore, 119228, Singapore; College of Intelligent Systems Science and Engineering, Harbin Engineering University, Harbin, 150001, China; Qingdao Innovation and Development Center, Harbin Engineering University, Qingdao, 266000, China; Sanya Nanhai Innovation and Development Base, Harbin Engineering University, Sanya, 572024, China; Stem Cell and Regenerative Biology, Genome Institute of Singapore, A*STAR, Singapore, 138672, Singapore; College of Intelligent Systems Science and Engineering, Harbin Engineering University, Harbin, 150001, China; Qingdao Innovation and Development Center, Harbin Engineering University, Qingdao, 266000, China; Sanya Nanhai Innovation and Development Base, Harbin Engineering University, Sanya, 572024, China; Department of Ophthalmology, Yong Loo Lin School of Medicine, National University of Singapore, Singapore, 119228, Singapore

## Abstract

**Motivation:**

Hypergraph-based models for brain disorder prediction mainly adopt imaging-derived hypergraphs as propagation backbones. However, the entanglement of topology construction and feature propagation leaves regional representations weakly constrained by underlying biological organization, making them vulnerable to subject-specific variation and noise, particularly in heterogeneous multimodal settings.

**Results:**

We present BriGHT, a **B**rain t**r**anscr**i**ptome-re**G**ularized **H**ypergraph framework for mul**T**imodal disorder prediction. BriGHT employs a transcriptome-derived structural reference as a soft anchoring prior to regularize neuroimaging ROI embeddings, stabilizing representation geometry while preserving disease-relevant subject-specific variation. BriGHT further incorporates a reliability-aware fusion module to estimate subject-specific modality reliability from prediction confidence, cross-modal consistency, and decision certainty, enabling adaptive integration under heterogeneous modality quality. Experiments on three neuroimaging cohorts (ADNI, ADHD-200, REST-meta-MDD) and four modalities (VBM, fMRI, FDG, AV45) demonstrate that BriGHT consistently outperforms competing graph/hypergraph learning methods across six brain disorder prediction tasks. Perturbation analyses show that BriGHT benefits from the spatial correspondence between transcriptomic modules and imaging ROIs, rather than from arbitrary hypergraph regularization alone. Ablation and meta-analytic interpretability analyses support the contribution of transcriptomic anchoring and adaptive fusion to robust and biologically meaningful brain disorder prediction.

**Availability:**

The software is publicly available at: https://github.com/Yaolab-fantastic/BriGHT.

## 1 Introduction

Brain disorder (BD) prediction from multimodal neuroimaging has become an important problem because different modalities provide complementary information about brain structure, function, and metabolism (Cong *et al.* 2024, Huang and Shu 2025). In this setting, graph learning has emerged as a natural framework for representing the brain as a network of regions of interest (ROIs) and for learning discriminative connectivity patterns from imaging data (Jiao *et al.* 2025). Among advances in network modeling, hypergraph-based methods show promise by extending pairwise graphs to higher-order relationships, enabling the modeling of group-wise ROI interactions (Feng *et al.* 2019, Han *et al.* 2024, Qiu *et al.* 2024).

Existing hypergraph learning for BD prediction falls into two groups. The first uses hypergraphs derived from neuroimaging as the propagation backbone, with strategies proposed to improve hypergraph construction (Qiu *et al.* 2024), weighting (Feng *et al.* 2019), aggregation (Han *et al.* 2024), or fusion (Yang *et al.* 2024, Luo *et al.* 2026). The second uses hypergraphs as structural regularizers, imposing hypergraph Laplacian or manifold penalties within feature selection, matrix factorization, or graph-regularized frameworks (Zhang *et al.* 2022, Ban *et al.* 2023, Wang *et al.* 2023).

Despite methodological differences, these approaches remain imaging-derived, as both the topology and representations are constructed from the same imaging sources. Therefore, regional embeddings are primarily shaped by modality-specific imaging relationships without explicit constraints from external biological organization. This lack of biological reference may reduce representation consistency across subjects and modalities, making embeddings vulnerable to subject-specific noise, acquisition artifacts, and modality variation (Fortin *et al.* 2018, Chen *et al.* 2025).

This problem becomes more severe in heterogeneous multimodal settings, where modality-specific distortions can be further amplified before multimodal fusion. Different modalities are typically processed through separate graph/hypergraph branches, so the resulting ROI embeddings inherit modality-specific noise patterns, structural biases, and quality variation (Cai *et al.* 2025, Yin *et al.* 2025). Therefore, homologous brain regions may be represented inconsistently not only across subjects but also across modalities, making the learned embeddings increasingly difficult to compare under a common functional basis. This weak cross-subject and cross-modality consistency may reduce the extent to which multimodal learning can recover stable disease-relevant organization from heterogeneous neuroimaging data.

We therefore propose BriGHT, a transcriptome-regularized framework that coordinates subject-specific representation learning with transcriptome-derived structural anchoring. Instead of using hypergraphs solely as propagation backbones, BriGHT introduces a shared biological prior as a soft anchor for subject- and modality-specific ROI representations. In our implementation, this anchor is instantiated from brain-wide transcriptomic organization and imposed through layer-wise higher-order regularization. Such anchoring does not enforce uniformity across subjects; instead, it stabilizes ROI-level representations around a shared higher-order organization while preserving disease-relevant individual deviations at the subject level. Building on these anchored representations, BriGHT further incorporates a reliability-aware fusion strategy that adaptively integrates multimodal information by jointly modeling prediction confidence, cross-modal consistency, and decision certainty under heterogeneous modality quality.

Our main contributions are summarized as follows:

- We introduce a biologically anchored multimodal framework named BriGHT for brain disorder prediction, where transcriptomic organization is incorporated as a structural reference for subject-specific neuroimaging representations via hypergraph regularization. By constraining ROI embeddings under a shared molecular structure, BriGHT improves cross-subject and cross-modality representation stability beyond imaging-derived variation.
- We develop a reliability-aware fusion strategy that integrates modality-specific representations by jointly modeling prediction confidence, cross-modal consistency, and decision certainty under heterogeneous modality quality.
- Extensive experiments on ADNI, ADHD-200, and REST-meta-MDD demonstrate that BriGHT consistently outperforms competing graph and hypergraph methods across six prediction tasks. Additional analyses confirm the robustness and biological interpretability of BriGHT.

## 2 Materials and methods

**Figure 1:**
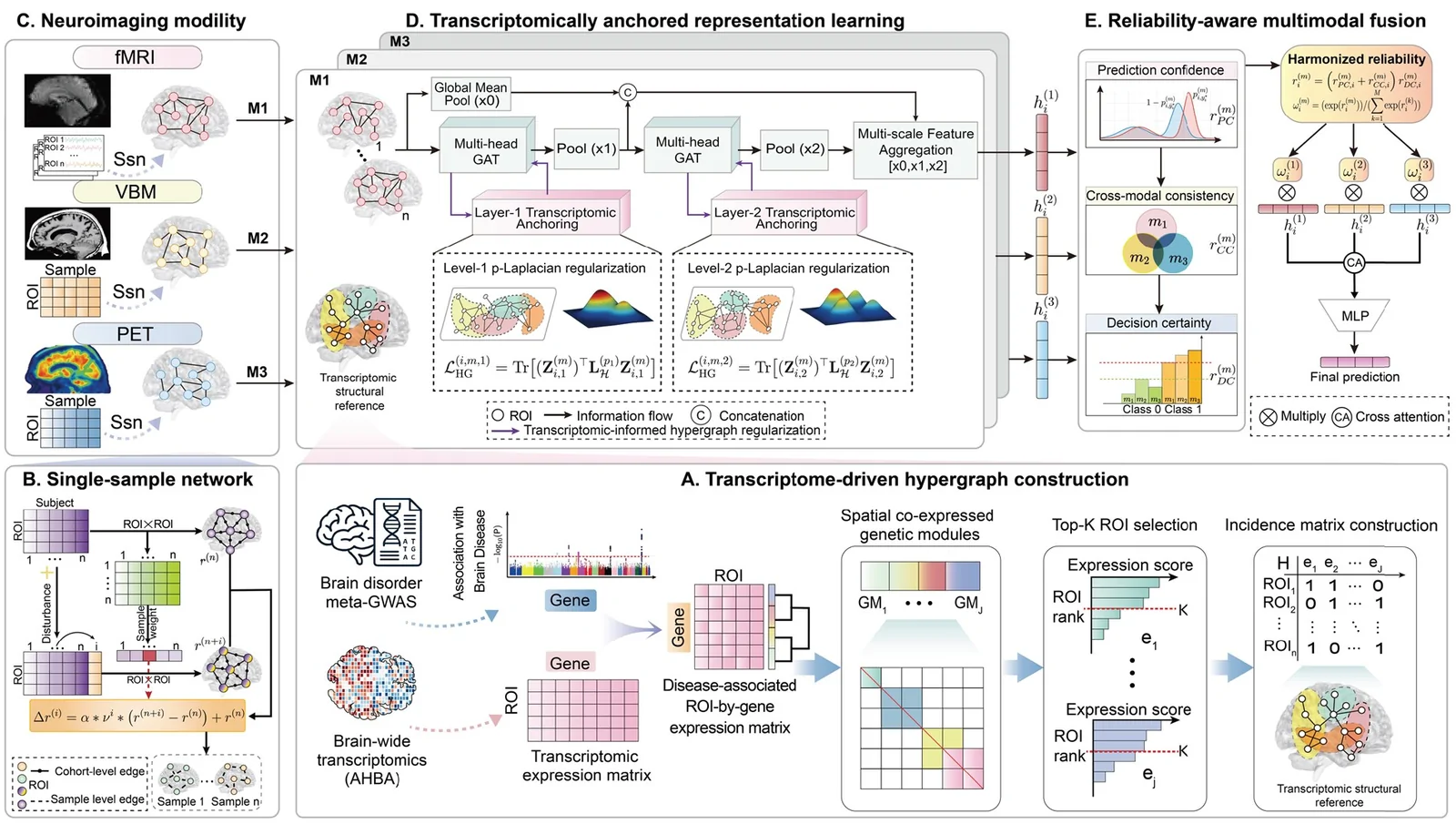
Overview of BriGHT. (A) GWAS-guided AHBA expression profiles are grouped into co-expression modules, which define an ROI hypergraph as a structural reference. (B) Subject-specific ROI networks are constructed for static imaging modalities using a single-sample strategy. (C) Multimodal imaging data are represented as modality-specific ROI graphs. (D) Transcriptomic-informed hypergraph regularization anchors ROI embeddings during graph learning, followed by multi-scale readout to obtain modality-specific subject embedding. (E) Reliability-aware fusion dynamically integrates modalities based on prediction confidence, cross-modal consistency, and decision certainty for final prediction.

An overview of BriGHT is shown in Fig. 1. Briefly, GWAS-guided transcriptomic modules are used to construct an ROI-level hypergraph, which serves as a structural constraint for learning subject-specific neuroimaging representations before reliability-aware multimodal fusion. Here, we summarize the common notation used throughout the study. Let *n* denote the number of subjects, *M* the number of imaging modalities, *d* the number of ROIs, *g* the number of disease-relevant genes, *J* the number of transcriptomic co-expression modules, and *L* the number of graph encoder layers. Subject and modality indices are denoted by *i* and *m*, respectively, while *v*, *r*, and *j* denote ROI, gene, and transcriptomic module indices.

### 2.1 Transcriptomic structural reference

To derive a disease-relevant structural reference, we leverage the Allen Human Brain Atlas (AHBA), which provides a genome-wide expression atlas across brain regions. Guided by meta-GWAS, we first retain significant genes and extract their matched expression profiles from AHBA, yielding a disease-associated ROI-by-gene expression matrix $B\in R^{d\times g}$ (Fig. 1A).

Rather than directly modeling individual genes or pairwise ROI similarities, we characterize co-expression across brain regions. Specifically, genes are grouped into co-expression modules according to their spatial expression similarity across ROIs.

We retain *J* modules to define the hypergraph prior. Each retained module therefore represents a disease-relevant molecular program with a coherent regional expression pattern. For each module, we compute an ROI-wise expression score by averaging the expression levels of its member genes. This yields a score vector $S_{j}\in R^{d}$, whose entries quantify the expression strength of each module across all ROIs.

To represent module-level molecular structure at the regional level, ROIs are ranked according to their score for each module, and the top-*K* ROIs are selected to form a hyperedge. Each hyperedge *e* therefore represents a set of brain regions jointly enriched for the same co-expression module. Collecting all hyperedges yields an ROI hypergraph H=(V,E) with $E={\left\{e_{j}\right\}}_{j=1}^{J}$. Its incidence matrix $H\in R^{d\times J}$ is defined by


(1) $$H_{v,j}=I(v\in e_{j}),$$

where I(·) denotes the indicator function. The ROI hypergraph H then serves as a structural reference for subsequent representation learning. See Supplementary Methods S1.2 for details.

### 2.2 Subject-specific ROI network

All imaging modalities are represented under a unified ROI-level graph formulation Fig. 1C. The graph associated with subject *i* and modality *m* is denoted as $G_{i}^{\left(m\right)}=(V,A_{i}^{\left(m\right)},X_{i}^{\left(m\right)})$, where *V* denotes the ROI set, $A_{i}^{\left(m\right)}$ denotes the adjacency matrix, and $X_{i}^{\left(m\right)}$ denotes the corresponding node feature matrix.

For static imaging modalities such as sMRI and PET, subject-specific connectivity is estimated using a cohort-informed single-sample network strategy (Chen *et al.* 2023) (Fig. 1B). The resulting connectivity matrices are then thresholded to obtain the adjacency matrix $A_{i}^{\left(m\right)}$, while the corresponding ROI measurements are used as node features. For dynamic modalities such as fMRI, subject-specific connectivity is directly estimated by Pearson correlation across ROI time series, yielding the adjacency matrix $A_{i}^{\left(m\right)}$. PCA-reduced temporal features were used as node attributes. Details are described in Supplementary Methods S1.3 and Table S1.

### 2.3 Transcriptomic anchoring

We implement transcriptomic anchoring through layer-wise hypergraph regularization on ROI-level embeddings (Fig. 1D). Given the ROI graph $G_{i}^{\left(m\right)}$ and the transcriptome-derived ROI hypergraph H, the goal is to learn modality-specific neuroimaging representations while constraining their ROI-level geometry under disorder-relevant molecular structure.

For subject *i* and modality *m*, the input node feature matrix is denoted as $Z_{i,0}^{\left(m\right)}=X_{i}^{\left(m\right)}$. Let $Z_{i,\ell }^{\left(m\right)}\in R^{d\times f_{\ell }}$ denote the embeddings at encoder layer ℓ, where $f_{\ell }$ is the embedding dimension. A graph encoder is applied to each subject-specific ROI graph to propagate imaging-derived information over the corresponding modality-specific topology. In implementation, the encoder is instantiated using graph attention layers. For ROI *v*, the layer-wise update is formulated as


(2) $$z_{i,\ell +1,v}^{\left(m\right)}=\phi \left(\sum_{u\in N_{i}^{\left(m\right)}(v)}\alpha_{i,\ell ,vu}^{\left(m\right)}W_{\ell }^{\left(m\right)}z_{i,\ell ,u}^{\left(m\right)}\right),$$

where $N_{i}^{\left(m\right)}(v)$ denotes the neighborhood of *v* in $G_{i}^{\left(m\right)}$, $\alpha_{i,\ell ,vu}^{\left(m\right)}$ is the attention coefficient from ROI *u* to *v*, $W_{\ell }^{\left(m\right)}$ is a learnable projection matrix, and ϕ(·) is a nonlinear activation function.

Using the incidence matrix H of the transcriptome-derived ROI hypergraph H defined in Section 2.1, we construct the corresponding hypergraph *p*-Laplacian at layer ℓ as


(3) $$L_{H}^{\left(p_{\ell }\right)}=I_{d}-D_{v}^{-p_{\ell }/2}H\Omega D_{e}^{-p_{\ell }}H^{⊤}D_{v}^{-p_{\ell }/2}.$$

Here, $D_{v}$ and $D_{e}$ denote the vertex-degree and hyperedge-degree matrices, respectively, Ω denotes the diagonal hyperedge weight matrix, and $p_{\ell }$ controls the strength of higher-order smoothing at layer ℓ. When $p_{\ell }=1$, this formulation reduces to generalized normalized hypergraph Laplacian. The layer-wise ROI anchoring loss is then defined as


(4) $$L_{\text{HG}}^{\left(i,m,\ell \right)}=\text{Tr}\left[{\left(Z_{i,\ell }^{\left(m\right)}\right)}^{⊤}L_{H}^{\left(p_{\ell }\right)}Z_{i,\ell }^{\left(m\right)}\right].$$

This term provides a soft structural constraint on the latent ROI embedding space by regularizing embeddings with respect to the transcriptomic hypergraph. Importantly, the regularization is imposed on ROI geometry within each subject and modality, rather than directly forcing different subjects to have identical embeddings. Therefore, the model preserves subject-specific imaging deviations while anchoring them under a common molecularly defined structure. Hyperparameter analysis of $p_{\ell }$ is shown in Fig. S2.

The total transcriptomic hypergraph regularization term is accumulated over subjects, modalities, and encoder layers:


(5) $$L_{\text{HG}}=\frac{1}{nM}\sum_{i=1}^{n}\sum_{m=1}^{M}\sum_{\ell =1}^{L}\lambda_{\ell }L_{\text{HG}}^{\left(i,m,\ell \right)},$$

where $\lambda_{\ell }$ controls the contribution of the constraint at layer ℓ.

After encoding, ROI-level embeddings are aggregated into subject representations via ROI-wise readout. For each subject and modality, readout features from all encoder layers are concatenated to form modality-specific representation $h_{i}^{\left(m\right)}$, thereby preserving multi-scale information across different propagation depths. The resulting representation is used as the modality-specific input for subsequent multimodal integration.

### 2.4 Reliability-aware multimodal fusion

To further address subject-wise variation in modality informativeness, we propose a harmonized reliability fusion strategy based on prediction confidence, cross-modal consistency, and decision certainty Fig. 1E. For each modality, a modality-specific classifier maps $h_{i}^{\left(m\right)}$ to class probabilities $p_{i}^{\left(m\right)}$.

#### 2.4.1 Prediction confidence

Given the representation $h_{i}^{\left(m\right)}$, we estimate its prediction confidence using a harmonized confidence criterion (Liang *et al.* 2024, Yao *et al.* 2024, Luo *et al.* 2026). Briefly, during training, a label-derived prediction confidence score is constructed as


(6) $$r_{\text{PC},i}^{(m)*}=\frac{2}{1/(p_{i,y_{i}^{*}}^{\left(m\right)}+\epsilon )+1/\left(1-p_{i,{\bar{y}}_{i}^{*}}^{\left(m\right)}+\epsilon \right)},$$

where $y_{i}^{*}$ is the ground-truth class, $p_{i,y_{i}^{*}}^{\left(m\right)}$ and $p_{i,{\bar{y}}_{i}^{*}}^{\left(m\right)}$ denote the probabilities assigned to the target and non-target classes respectively, and ϵ is a small constant. Since the ground-truth label is unavailable during inference, we use two confidence networks to approximate $r_{\text{PC},i}^{(m)*}$. These networks are optimized by aligning the predicted score with the label-derived one:


(7) $$L_{\text{Conf}}=\frac{1}{Mn}\sum_{m=1}^{M}\sum_{i=1}^{n}{\left\|r_{\text{PC},i}^{\left(m\right)}-r_{\text{PC},i}^{(m)*}\right\|}^{2}+\frac{1}{M}\sum_{m=1}^{M}L_{\text{Cls}}^{\left(m\right)}.$$

Here, $r_{\text{PC},i}^{\left(m\right)}$ and $r_{\text{PC},i}^{(m)*}$ denote the estimated and label-derived confidence score, respectively, and $L_{\text{Cls}}^{\left(m\right)}$ is the modality-specific classification loss. During inference, prediction confidence is obtained directly from $h_{i}^{\left(m\right)}$ without ground-truth labels.

#### 2.4.2 Cross-modal consistency

We measure cross-modal consistency by evaluating the agreement between the prediction distribution of one modality and those of the remaining:


(8) $$r_{\text{CC},i}^{\left(m\right)}=\text{exp}\;\left(-\frac{1}{M-1}\sum_{k\neq m}{\left\|p_{i}^{\left(m\right)}-p_{i}^{\left(k\right)}\right\|}_{2}\right).$$

This score increases when the prediction of modality *m* is consistent with the other modalities and decreases otherwise.

#### 2.4.3 Decision certainty

We quantify decision certainty by measuring the sharpness of the predicted class distribution:


(9) $$r_{\text{DC},i}^{\left(m\right)}=1+\frac{1}{\;\text{log}\;C}\sum_{c=1}^{C}p_{i,c}^{\left(m\right)}\;\text{log}\;p_{i,c}^{\left(m\right)}.$$

This score ranges from 0 to 1, with larger values indicating sharper and more decisive predictions and smaller values indicating more uncertain class distributions.

#### 2.4.4 Harmonized reliability

We aggregate the above three reliability components into a unified reliability score. Prediction confidence and cross-modal consistency provide two complementary sources of positive evidence, while decision certainty acts as an uncertainty gate that attenuates ambiguous predictions. The reliability score for modality *m* is therefore defined as:


(10) $$r_{i}^{\left(m\right)}=\left(r_{\text{PC},i}^{\left(m\right)}+r_{\text{CC},i}^{\left(m\right)}\right)r_{\text{DC},i}^{\left(m\right)}.$$

The resulting scores are then converted into relative modality weights using softmax normalization:


(11) $$\omega_{i}^{\left(m\right)}=\text{exp}(r_{i}^{\left(m\right)})/\sum_{k=1}^{M}\;\text{exp}\;(r_{i}^{\left(k\right)}).$$

The weight $\omega_{i}^{\left(m\right)}$ is used to dynamically modulate the contribution of each modality during fusion. Reliability-weighted representations are then fused by cross-attention, yielding the multimodal representation $u_{i}$ for final prediction. Additional analysis is described in Supplementary Results S2.4.

### 2.5 Optimization objective

BriGHT is trained end-to-end by jointly optimizing prediction and transcriptomic structural anchoring:


(12) $$L=L_{\text{cls}}+\lambda L_{\text{HG}}+\eta L_{\text{Conf}}.$$

Here, $L_{\text{cls}}$ is the cross-entropy loss of the final multimodal classifier, $L_{\text{HG}}$ is the transcriptomic hypergraph regularization term, $L_{\text{Conf}}$ is the confidence estimation loss. λ and η control their relative contributions (Supplementary Results S2.3 and Fig. S3).

## 3 Results

### 3.1 Datasets and experimental settings

We use one transcriptomic reference dataset and three neuroimaging cohorts, with all data mapped to 116 AAL ROIs (Luo *et al.* 2026). Details are described below, as well as provided in Table 1 and Supplementary Methods S1.1.

**Table 1:** Summary of datasets used in this study.

Dataset	Modality	**Subject**	Gender (M/F)	Age (mean ± SD)
ADNI	AV45 FDG VBM	NC:221	114/107	76.34 ± 6.58
		EMCI:275	155/120	71.50 ± 7.12
		LMCI:190	111/79	74.08 ± 8.55
		AD:165	99/66	75.35 ± 7.88
		pMCI:69	35/34	72.89 ± 8.26
		sMCI:116	74/42	75.09 ± 8.44
ADHD-200	fMRI VBM	NC:101	48/53	12.34 ± 3.08
		ADHD:93	75/18	10.49 ± 2.48
REST- meta-MDD	fMRI VBM	NC:771	316/455	34.55 ± 13.14
		MDD:830	306/524	34.39 ± 11.57

#### 3.1.1 AHBA transcriptomic reference

Transcriptomic data were obtained from the Allen Human Brain Atlas (AHBA), and regional expression profiles were derived using the abagen toolbox (Markello *et al.* 2021), yielding 15 633 genes across the brain. To construct disorder-relevant transcriptomic structural references, we incorporated evidence from meta-GWAS studies (Demontis *et al.* 2019, Howard *et al.* 2019, Kunkle *et al.* 2019) and retained genes associated with each disorder (Supplementary S2.1, Fig. S6), resulting in 1216 genes for AD, 1524 genes for ADHD, and 2463 genes for MDD. These genes were used to derive co-expression modules and module-defined ROI hypergraphs.

#### 3.1.2 ADNI cohort

Multimodal neuroimaging data for Alzheimer’s disease analysis were obtained from the Alzheimer’s Disease Neuroimaging Initiative (ADNI). The dataset included AV45, FDG, and VBM, with diagnostic labels of NC, EMCI, LMCI, and AD; EMCI and LMCI were combined as MCI in the three-class classification task. A total of 851 participants were included. We further divided LMCIs into progressive MCI (pMCI) and stable MCI (sMCI) according to 36-month longitudinal conversion status; 5 LMCI patients who reverted to NC were excluded. All scans were preprocessed following (Yao *et al.* 2020).

#### 3.1.3 ADHD-200 cohort

We used data from the New York University site (NYU) of ADHD-200, including sMRI and resting-state fMRI (rs-fMRI) from 194 participants (101 NCs; 93 ADHDs). Imaging data were processed (Yao *et al.* 2020) and ROI measurements were extracted based on the AAL atlas.

#### 3.1.4 REST-meta-MDD cohort

The REST-meta-MDD dataset was included as a large-scale multi-site psychiatric cohort for MDD classification. Following the curated cohort (Yan *et al.* 2019), we used 1601 participants (771 NCs; 830 MDDs). ROI-level measurements were extracted from both VBM and fMRI.

#### 3.1.5 Benchmark methods

We compared BriGHT with 10 methods. The baselines included three GNNs (GCN, GAT, GraphSAGE) and two hypergraph models (HGNN and HyperGCN). We further included five advanced graph learning methods, including Cross-GNN (TMI’23) (Yang *et al.* 2024) with cross-graph convolution, EV-GCN (MIA’22) (Huang and Chung 2022) with edge-variational population graph learning, MMGL (TMI’22) (Zheng *et al.* 2022) with adaptive graph construction, AL-NEGAT (TNNLS’24) (Chen *et al.* 2024) with adversarial node–edge graph attention, and MM-GTUNets (TMI’25) (Cai *et al.* 2025) with graph transformer.

#### 3.1.6 Evaluation metrics

Model performance was evaluated using accuracy (ACC) and the area under the ROC curve (AUC) for binary classification, with macro F1 (F1-m) reported for the multi-class task. Mean ± SD from five-fold cross-validation is reported. Significance was assessed by paired *t*-tests.

#### 3.1.7 Implementation details

We implemented the model in PyTorch 2.1.0 and optimized using Adam. Models were trained for 3000 epochs with an initial learning rate of $1\times 10^{-3}$ and a weight decay of $1\times 10^{-4}$. All experiments were conducted on an NVIDIA RTX 4090 GPU with 24 GB of memory.

### 3.2 Performance of BriGHT

Table 2 reports the performance of BriGHT against competing methods across six tasks. BriGHT achieves the highest values for all reported metrics, indicating stable performance across diagnostic, progression-related, and cross-disorder classification settings. On ADNI, the gains are not confined to the relatively separable NC versus AD task, where ACC increases from 96.6% to 98.6%, but also extend to more challenging early-stage, progression, and multi-class staging predictions. Specifically, BriGHT improves ACC from 76.4% to 78.9% for NC versus EMCI, from 87.5% to 89.2% for pMCI versus sMCI, and from 77.1% to 80.6% for NC versus MCI versus AD.

**Table 2:** Comparison with state-of-the-art methods across multiple tasks on the ADNI, ADHD-200, and REST-meta-MDD datasets.

Method	NC versus AD		NC versus EMCI		pMCI versus sMCI		NC versus MCI versus AD		NC versus ADHD		NC versus MDD	
	ACC	AUC	ACC	AUC	ACC	AUC	ACC	F1-m	ACC	AUC	ACC	AUC
GCN	86.4 ± 2.6	86.3 ± 2.5	70.1 ± 2.0	71.2 ± 1.9	80.5 ± 2.6	81.4 ± 2.3	71.2 ± 2.9	68.4 ± 2.2	71.2 ± 2.3	71.6 ± 1.8	62.2 ± 2.6	68.5 ± 2.5
GAT	92.4 ± 0.9	93.1 ± 0.8	74.2 ± 1.1	73.8 ± 1.1	83.5 ± 1.1	83.3 ± 1.2	73.0 ± 1.2	73.5 ± 1.5	74.3 ± 1.0	74.5 ± 1.0	65.8 ± 1.2	66.0 ± 1.2
GraphSAGE	88.4 ± 1.8	87.5 ± 2.0	71.5 ± 2.4	72.3 ± 2.3	79.8 ± 1.8	80.5 ± 2.1	70.5 ± 2.1	70.8 ± 2.4	70.5 ± 1.6	70.1 ± 1.5	61.8 ± 1.5	64.1 ± 1.6
HGNN	93.1 ± 1.9	93.5 ± 1.4	73.8 ± 1.0	74.9 ± 0.9	83.8 ± 1.5	84.1 ± 1.5	73.2 ± 1.3	72.9 ± 1.4	74.1 ± 0.9	75.4 ± 0.8	66.5 ± 1.3	68.8 ± 1.4
HyperGCN	92.5 ± 0.7	93.2 ± 0.6	72.2 ± 1.2	71.2 ± 1.2	82.5 ± 1.3	83.9 ± 1.4	72.5 ± 1.6	71.1 ± 1.9	73.9 ± 1.6	73.1 ± 1.1	64.9 ± 1.5	67.1 ± 1.5
Cross-GNN	93.8 ± 1.7	94.2 ± 1.3	74.5 ± 1.3	74.1 ± 1.1	84.2 ± 1.4	85.1 ± 1.3	76.4 ± 1.6	77.5 ± 1.8	75.8 ± 1.3	74.9 ± 1.1	68.0 ± 1.4	71.5 ± 1.3
EV-GCN	96.1 ± 0.8	96.8 ± 0.7	76.2 ± 0.9	76.8 ± 0.8	86.9 ± 1.0	87.9 ± 1.0	77.1 ± 1.0	77.6 ± 1.2	77.8 ± 1.2	77.9 ± 1.0	70.5 ± 0.9	74.1 ± 0.8
MMGL	95.5 ± 0.9	96.2 ± 0.8	75.9 ± 1.1	75.1 ± 1.0	85.1 ± 1.2	86.5 ± 0.8	75.8 ± 1.3	76.4 ± 1.5	76.0 ± 1.1	76.2 ± 0.9	68.3 ± 1.2	71.8 ± 1.1
AL-NEGAT	94.1 ± 2.1	94.7 ± 1.9	73.8 ± 1.8	73.4 ± 1.6	84.6 ± 1.7	85.6 ± 1.6	73.4 ± 2.1	74.2 ± 2.2	74.6 ± 1.6	74.6 ± 1.3	67.7 ± 1.8	69.1 ± 1.7
MM-GTUNets	96.6 ± 0.7	97.2 ± 1.0	76.4 ± 1.2	76.5 ± 1.0	87.5 ± 0.9	87.5 ± 1.2	76.6 ± 1.4	76.4 ± 1.6	78.3 ± 1.5	78.6 ± 1.6	70.6 ± 1.1	74.3 ± 1.2
**BriGHT (Ours)**	98.6 ± 0.8*	98.2 ± 0.4*	78.9 ± 0.9*	**77.4 ± 1.2**	89.2 ± 1.6*	90.1 ± 1.8*	80.6 ± 2.1*	**79.4 ± 2.3**	81.3 ± 1.1*	81.5 ± 1.5*	73.4 ± 1.4*	76.9 ± 1.3*

**Bold**: best performance. Underlined: second-best performance.

* Indicates statistically significant superiority (paired *t*-test, *P* < .05).

Consistent improvements are also observed in the ADHD-200 and REST-meta-MDD cohorts. For example, BriGHT improves ACC from 78.3% to 81.3% on ADHD-200 and from 70.6% to 73.4% on REST-meta-MDD, compared with the best competing methods. These results indicate that the advantage of BriGHT extends to disorder categories, heterogeneous cohorts and imaging modalities, suggesting that the learned multimodal representations remain stable under diverse clinical settings.

### 3.3 Ablation and modality contribution

We performed ablation experiments to evaluate the effects of transcriptomic anchoring and reliability-aware fusion by removing each component from the full model. As shown in Table 3, removing either transcriptomic regularization or reliability fusion decreases performance across all tasks, while removing both causes the largest drop. This suggests that biological priors and adaptive modality weighting provide complementary benefits. Robustness analyses of these modules are provided in Supplementary Results S2.5 and Fig. S4A, B.

**Table 3:** Ablation study of transcriptomic anchoring (TA) and reliability-aware fusion (RF).

TA	RF	NC versus AD		NC versus EMCI		pMCI versus sMCI		NC versus MCI versus AD		NC versus ADHD		NC versus MDD	
		ACC	AUC	ACC	AUC	ACC	AUC	ACC	F1-m	ACC	AUC	ACC	AUC
**✓**	✓	**98.6** ± **0.8**	**98.2** ± **0.4**	**78.9** ± **0.9**	**77.4** ± **1.2**	**89.2** ± **1.6**	**90.1** ± **1.8**	**80.6** ± **2.1**	**79.4** ± **2.3**	**81.3** ± **1.1**	**81.5** ± **1.5**	**73.4** ± **1.4**	**76.9** ± **1.3**
**✓**	✗	97.4 ± 1.0	97.0 ± 0.6	77.6 ± 1.1	76.2 ± 1.3	88.1 ± 1.8	88.5 ± 2.0	79.2 ± 2.3	78.1 ± 2.5	79.9 ± 1.3	80.2 ± 1.6	72.1 ± 1.5	75.6 ± 1.4
**✗**	✓	97.9 ± 1.0	97.5 ± 0.6	77.5 ± 1.3	76.1 ± 1.5	87.8 ± 2.0	88.6 ± 2.2	78.4 ± 2.3	77.2 ± 2.5	79.5 ± 1.4	79.2 ± 1.7	70.9 ± 1.7	74.2 ± 1.6
**✗**	✗	96.2 ± 1.4	95.9 ± 1.3	76.2 ± 1.6	74.8 ± 1.8	86.5 ± 2.2	87.2 ± 2.4	77.2 ± 2.6	75.9 ± 2.7	78.3 ± 1.8	78.1 ± 2.5	68.2 ± 2.5	70.8 ± 2.8

Best results are in **bold**.

**Figure 2:**
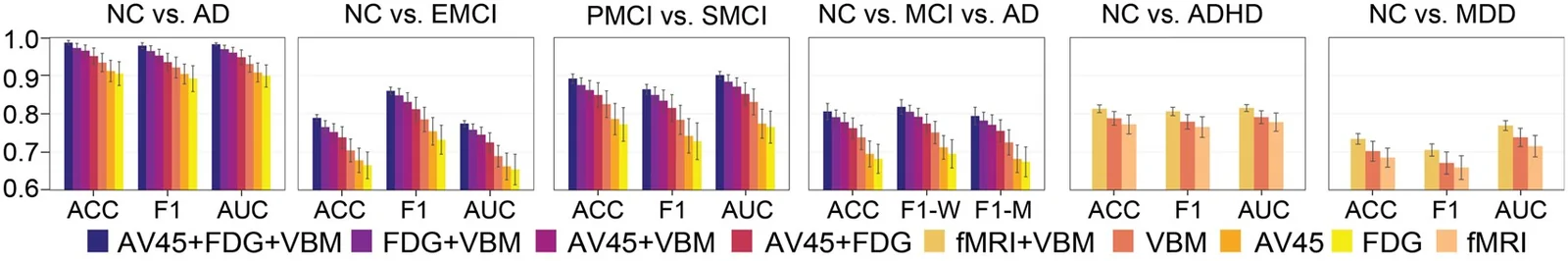
Performance comparison under different modality combinations across tasks.

Figure 2 further shows that the most informative modality subsets vary across disorder categories. In AD-related cognitive impairment tasks, VBM alone and VBM combined with other PET modalities generally outperform PET-only settings, suggesting that structural atrophy patterns provide stable discriminative information for AD/MCI classification. In contrast, fMRI outperforms VBM in ADHD and MDD classification, indicating that functional alterations may provide more sensitive information for these two disorders.

### 3.4 Transcriptomic anchoring

To examine the biological specificity of the proposed transcriptomic anchoring, we evaluated three perturbed hypergraph settings for regularization: (i) module-preserved random ROI assignment, where each module was assigned a random set of *K* ROIs instead of the top *K*; (ii) degree-matched randomization, where hyperedges were randomized while preserving ROI degrees and hyperedge sizes; and (iii) shuffled ROI-module mapping, where ROI indices were randomly permuted when applying the original incidence matrix. For reference, we also report a no-transcriptomic anchoring setting in which hypergraph regularization is removed.

Table 4 shows that our original transcriptome-derived hypergraph performs best across all tasks and metrics. Module-preserved random ROI remains competitive, suggesting that high-order regularization provides some structural smoothing, whereas degree-matched randomization performs close to or below the no-anchoring setting, indicating that coarse hypergraph statistics alone are insufficient. The largest drop occurs with shuffled ROI-module mapping, suggesting that anatomically mismatched transcriptomic structure can introduce misleading constraints.

**Table 4:** Biological specificity analysis of transcriptomic anchoring.

Regularization perturbation	NC versus AD		NC versus EMCI		pMCI versus sMCI		NC versus MCI versus AD		NC versus ADHD		NC versus MDD	
	ACC (%)	AUC (%)	ACC (%)	AUC (%)	ACC (%)	AUC (%)	ACC (%)	F1-m (%)	ACC (%)	AUC (%)	ACC (%)	AUC (%)
**BriGHT (ours)**	**98.6** ± **0.8**	**98.2** ± **0.4**	**78.9** ± **0.9**	**77.4** ± **1.2**	**89.2** ± **1.6**	**90.1** ± **1.8**	**80.6** ± **2.1**	**79.4** ± **2.3**	**81.3** ± **1.1**	**81.5** ± **1.5**	**73.4** ± **1.4**	**76.9** ± **1.3**
No transcriptomic anchoring	97.9 ± 1.0	97.5 ± 0.6	77.5 ± 1.3	76.1 ± 1.5	87.8 ± 1.8	88.6 ± 2.2	78.4 ± 2.3	77.2 ± 2.5	79.5 ± 1.4	79.2 ± 1.7	70.9 ± 1.7	74.2 ± 1.6
Module-preserved random ROI	98.1 ± 1.0	97.7 ± 0.7	77.8 ± 1.2	76.6 ± 1.5	88.4 ± 1.8	89.2 ± 2.1	79.2 ± 2.4	78.1 ± 2.6	80.3 ± 1.3	80.2 ± 1.8	72.0 ± 1.7	75.4 ± 1.6
Degree-matched randomization	97.7 ± 1.2	97.2 ± 0.8	77.2 ± 1.4	75.8 ± 1.7	87.5 ± 2.1	88.2 ± 2.4	78.1 ± 2.6	76.8 ± 2.8	79.1 ± 1.6	78.8 ± 2.0	70.4 ± 2.0	73.8 ± 1.9
Shuffled ROI-module mapping	97.3 ± 1.4	96.8 ± 1.0	76.5 ± 1.6	75.1 ± 1.8	86.9 ± 2.4	87.5 ± 2.7	77.4 ± 2.9	76.1 ± 3.0	78.5 ± 1.8	78.2 ± 2.2	69.8 ± 2.3	73.2 ± 2.3

Best are in **bold**.

Our perturbation results indicate that transcriptomic anchoring does not improve performance simply by adding an arbitrary hypergraph regularizer. Its benefit depends on the spatial correspondence between biologically functional modules and brain regions, supporting the biological specificity of the transcriptome-derived structural anchor.

### 3.5 Biological interpretability

#### 3.5.1 Brain biomarkers

**Figure 3:**
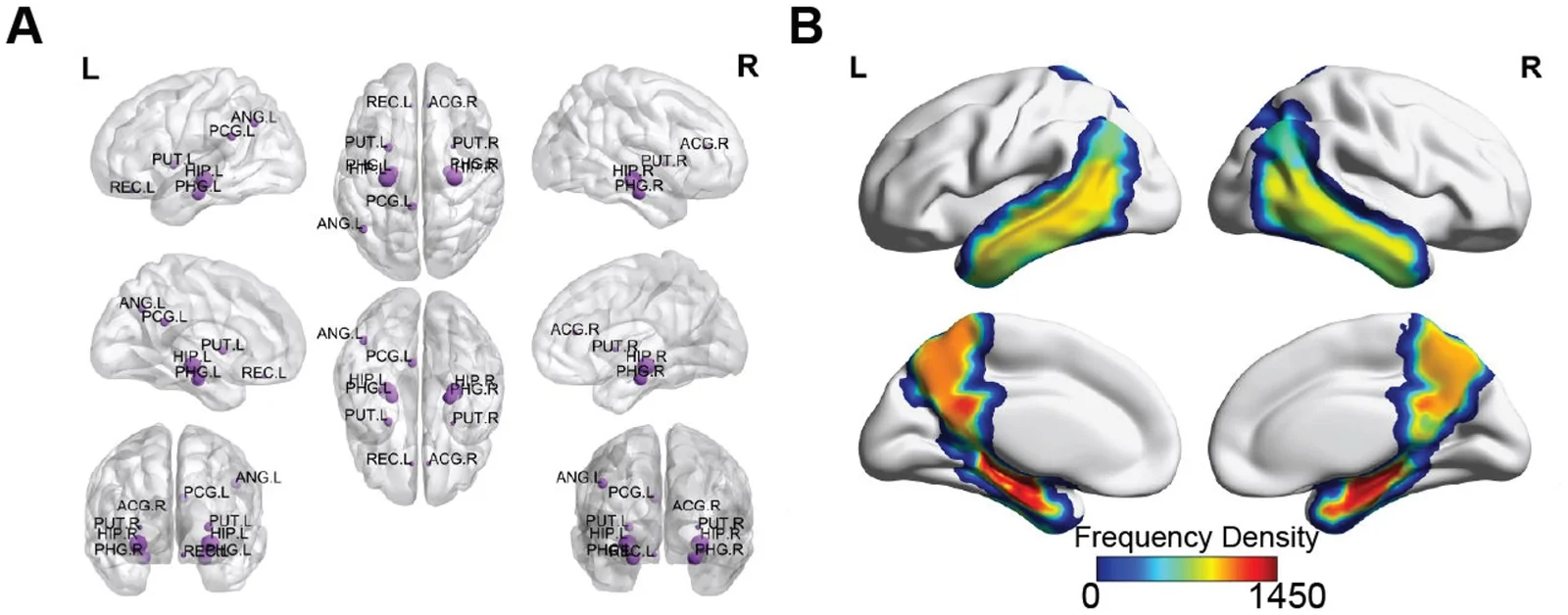
(A) Top 10 discriminative regions from NC versus AD and (B) their literature-derived functional relevance from NeuroQuery.

Figure 3A and Fig. S5A (Supplementary S2.6) visualize the top-10 ROIs identified by feature ablation. For AD-related tasks, the highlighted regions are mainly located in the temporal and parietal cortices, which are closely associated with memory impairment and neurodegeneration. For ADHD and MDD classification, the top ROIs are mainly distributed in frontal and limbic systems, reflecting disruptions in attention control and emotional regulation. These patterns suggest that BriGHT captures disease-relevant regional information, rather than purely dataset-specific discriminative cues.

#### 3.5.2 Functional relevance

We further assessed the functional relevance of the top-ranked ROIs using large-scale neuroimaging meta-analysis. Specifically, task-specific frequency density maps were derived with NiMARE (Salo *et al.* 2022) based on NeuroQuery (Dockès *et al.* 2020), which aggregates activation patterns from 13 459 neuroimaging studies. As shown in Fig. 3B and Fig. S5B, top ROIs show spatial correspondence with high-frequency areas in the corresponding meta-analytic maps across tasks. This agreement supports the functional relevance of the identified discriminative regions.

## 4 Discussion and conclusions

We proposed BriGHT, a biologically anchored multimodal learning framework for brain disorder prediction. Different from graph or hypergraph learning approaches that derive both topology and representations from neuroimaging data alone, BriGHT introduces transcriptomic organization as a structural anchor for ROI-level representation learning. By integrating GWAS-guided transcriptome priors, subject-specific imaging graphs, and reliability-aware multimodal fusion, BriGHT provides a cross-scale strategy for linking molecular organization with macroscale imaging representations.

The perturbation experiments clarify the source of the benefit from transcriptomic anchoring. When the ROI-module organization was randomized or anatomically mismatched, the performance advantage was reduced, indicating that the transcriptome-derived regularization contributes through its spatially organized molecular structure rather than through arbitrary topological constraints. This result is consistent with the design of BriGHT, in which transcriptomic organization serves as a biologically grounded reference for ROI-level representation learning, while subject-specific imaging deviations remain available for disease discrimination.

The multimodal results indicate that modality informativeness is task- and subject-dependent. Because structural and functional imaging capture distinct aspects of brain disorders, their contributions can vary across cohorts, disorders, and subjects. This supports the use of dynamic fusion, which adaptively weights modality-specific representations. Together with transcriptomic anchoring, this design allows BriGHT to learn representations that are biologically constrained while remaining flexible to heterogeneous imaging signals.

The current findings should be interpreted in light of available molecular and imaging resources. The AHBA-derived reference provides a spatial molecular organization map, but it is limited by donor number, sampling density, and lack of disease-state specificity. GWAS-guided co-expression module construction also depends on the quality and resolution of available statistics. Future work could incorporate larger molecular atlases, single-cell or spatial multiomics, longitudinal cohorts, and independent validation datasets to improve the biological resolution and generalizability of biologically anchored prediction models.

## Supplementary Material

btag681_Supplementary_Data

## Data Availability

The data underlying this article are available at https://github.com/Yaolab-fantastic/BriGHT. The ADNI data can be accessed at https://adni.loni.usc.edu; the ADHD-200 data can be accessed at http://fcon 1000.projects.nitrc.org/indi/adhd200; and the MDD data can be accessed at http://rfmri.org/REST-meta-MDD.
