# Supplementary material for: BriGHT: transcriptome-regularized multimodal neuroimaging for brain disorder prediction
Source: Bioinformatics. 2026 Sep 11;42(9):btag681. doi: 10.1093/bioinformatics/btag681 (PMC13613068; doi:10.1093/bioinformatics/btag681)
Supplement: Supplementary file 1 [file btag681_supplementary_data.pdf]

Supplemental Information for "BriGHT: transcriptome-regularized multimodal neuroimaging for brain disorder prediction"

Zhoujie Fan, Haoran Luo, Huilong Zhou, Hao Meng, Hong Liang,
Zheng Wang, Huazhu Fu, Ching-Yu Cheng, Shan Cong*, Xiaohui Yao*

S1 Supplementary Methods

S1.1 Transcriptomic data acquisition and processing

Brain-wide transcriptomic data were obtained from the Allen Human Brain Atlas (AHBA, `human.brain-map.org`), which contains more than 58,000 probes sampled from 3,702 brain tissue samples from six donors. We employed the *abagen* toolbox[1] for preprocessing and mapped gene expression profiles to 116 brain regions defined by the AAL atlas, resulting in 15,633 genes. To focus on disorder-relevant molecular signals, we used large-scale meta-GWAS summary statistics for AD, ADHD, and MDD. Specifically, we utilized summary statistics from Alzheimer's disease (AD)[2], ADHD[3], and MDD-related GWAS studies[4]. Gene-level p-values were computed from SNP-level statistics using MAGMA[5], and genes with nominal significance ($p < 0.05$) were retained. This resulted in 1,216 genes for AD, 1,524 genes for ADHD, and 2,463 genes for MDD, all mapped to the same 116 ROIs. These disease-specific gene sets were subsequently used to construct disorder-specific transcriptome-derived ROI hypergraphs.

S1.2 Construction of transcriptomic structural reference

For each disorder, genes with a gene-level association of $P < 0.05$ were selected from the corresponding GWAS summary statistics and mapped to the processed AHBA ROI-by-gene expression matrix. Genes in the lowest 10% of expression variance across ROIs were removed, and the remaining genes were standardized across ROIs before WGCNA module extraction. A signed co-expression network was constructed using biweight midcorrelation, with the soft-thresholding power selected from 1 to 29 according to the scale-free topology fit. Gene modules were identified using average-linkage hierarchical clustering and dynamic tree cutting, followed by module merging. Grey and undersized modules were excluded.

Then, the co-expression modules were used to construct a disorder-specific ROI hypergraph. For each module, the standardized expression values of its member genes were averaged to obtain an ROI-level module score, and the top K ROIs with the highest scores were connected as one hyperedge. The resulting incidence matrix was defined as $H_{v,j} = 1$ if ROI v belonged to hyperedge e_j, and $H_{v,j} = 0$ otherwise, with unit hyperedge weights $\Omega = I$. The resulting disorder-specific hypergraph was used as the transcriptomic structural reference in BriGHT.

S1.3 Neuroimaging data acquisition and preprocessing

Neuroimaging data were obtained from three brain disorder cohorts, including ADNI, ADHD-200, and REST-meta-MDD. Four imaging modalities were used across these cohorts: AV45-PET, FDG-PET, VBM-based structural MRI (VBM-sMRI), and resting-state fMRI (rs-fMRI).

For ADNI, preprocessed AV45-PET and FDG-PET scans were obtained from the ADNI database. Standard PET preprocessing included motion correction, spatial normalization, smoothing, and intensity normalization[6,7]. AV45-PET images were normalized to cerebellar gray matter, and regional SUVR values were extracted using the AAL atlas and MarsBaR toolbox[8]. FDG-PET scans were aligned to subject-specific T1-weighted MRI and normalized to MNI space, with regional glucose metabolism values extracted from AAL ROIs[9].

Structural MRI data were processed using voxel-based morphometry implemented in SPM12. T1-weighted images were normalized to MNI space, resampled, and smoothed with a Gaussian kernel. Gray matter density values were extracted from AAL ROIs and used as VBM-sMRI regional features[8]. The same VBM preprocessing strategy was applied to the cohorts where structural MRI was used.

Resting-state fMRI data from ADHD-200 and REST-meta-MDD were preprocessed using DPABI[10]. The preprocessing steps included removal of the first 10 volumes, slice timing correction, motion correction, spatial normalization, and smoothing. Subjects with excessive head motion were excluded. ROI-wise time series were extracted, and functional connectivity matrices were constructed using Pearson correlations between ROI time series. These correlation matrices were thresholded to obtain binary adjacency matrices. To construct compact node features, principal component analysis was applied to ROI-wise temporal signals, and the first principal component was retained as the representative feature. This choice was motivated by prior studies showing that dominant low-rank components can capture discriminative information[11,12]. As shown in Table S1, using the first principal component provided the best performance in this setting while reducing computational cost; therefore, this setting was used in all experiments.

Table S1: PCA component selections for fMRI ROI graph construction.

Number of PCs	NC vs. ADHD			
	ACC (%)	F1 (%)	AUC (%)	Time (s/epoch)
1	80.2±1.3	79.3±1.4	80.1±1.8	0.54
10	74.1±1.3	72.7±1.6	78.7±1.1	1.08
20	77.5±1.5	76.3±1.6	75.7±1.3	1.40
50	75.8±2.1	75.8±1.8	72.2±1.9	1.61

S2 Supplemental Results

S2.1 GWAS threshold sensitivity analysis

We evaluated the effect of the GWAS threshold on transcriptomic hypergraph construction and downstream prediction performance. Three thresholds, namely $P < 10^{-5}$, $P < 10^{-3}$, and $P < 0.05$, were examined. For each disorder and threshold, we constructed the corresponding ROI-by-gene expression matrix and generated the transcriptomic hypergraph using the same WGCNA-based construction procedure.

Table S2 summarizes the number of selected genes, the number of co-expression modules, the number of ROIs per hyperedge, ROI coverage, while Table S3 compares the corresponding model performance. We can observe that although the number of co-expression modules and ROIs per hyperedge varied across GWAS thresholds, the downstream prediction performance remained relatively stable once sufficient genes were retained for module construction. We also observed that including more candidate disease-associated genes generally led to better performance. This suggests that including more candidate genes may provide more comprehensive disease-related biological priors for constructing the structural reference. Therefore, we adopted $P < 0.05$ in our experiments.

Table S2: Number of genes used for hypergraph construction under different GWAS thresholds.

**Disorder**	**GWAS threshold**	**# of genes**	$\|J\|$	K	**ROI coverage**[a]
AD	$P < 1\text{E}-5$	37	2	30	64
	$P < 1\text{E}-3$	98	5	30	80
	$P < 0.05$	1094	5	25	93
ADHD	$P < 1\text{E}-5$	17	1	25	25
	$P < 1\text{E}-3$	139	4	25	73
	$P < 0.05$	1371	5	25	74
MDD	$P < 1\text{E}-5$	103	3	40	87
	$P < 1\text{E}-3$	1047	5	35	106
	$P < 0.05$	2216	5	30	112

[a] "ROI coverage" denotes the number of unique ROIs included in at least one hyperedge.

Table S3: Prediction performance under different GWAS significance thresholds.

GWAS threshold	NC vs. AD		NC vs. ADHD		NC vs. MDD	
	ACC (%)	AUC (%)	ACC (%)	AUC (%)	ACC (%)	AUC (%)
$P <$ 1E $- 5$	98.0 ± 1.2	97.5 ± 1.1	79.6 ± 1.4	79.5 ± 1.6	72.4 ± 1.7	75.7 ± 1.4
$P <$ 1E $- 3$	98.2 ± 1.0	97.7 ± 0.5	80.7 ± 1.3	80.9 ± 1.5	73.1 ± 1.5	76.3 ± 1.5
$P < 0.05$	$\mathbf{98.6 \pm 0.8}$	$\mathbf{98.2 \pm 0.4}$	$\mathbf{81.3 \pm 1.1}$	$\mathbf{81.5 \pm 1.5}$	$\mathbf{73.4 \pm 1.4}$	$\mathbf{76.9 \pm 1.3}$

S2.2 Hyperparameter sensitivity analysis

S2.2.1 Number of modules J and selected ROIs K

We assessed the sensitivity of BriGHT to key hyperparameters used in hypergraph construction, including the number of co-expression modules J and the number of selected ROIs per hyperedge K. As shown in Fig. S1, BriGHT maintains stable performance across a broad range of parameter settings, and the selected configuration lies within a high-performing region. These results indicate that the framework is robust to hyperparameter choices.

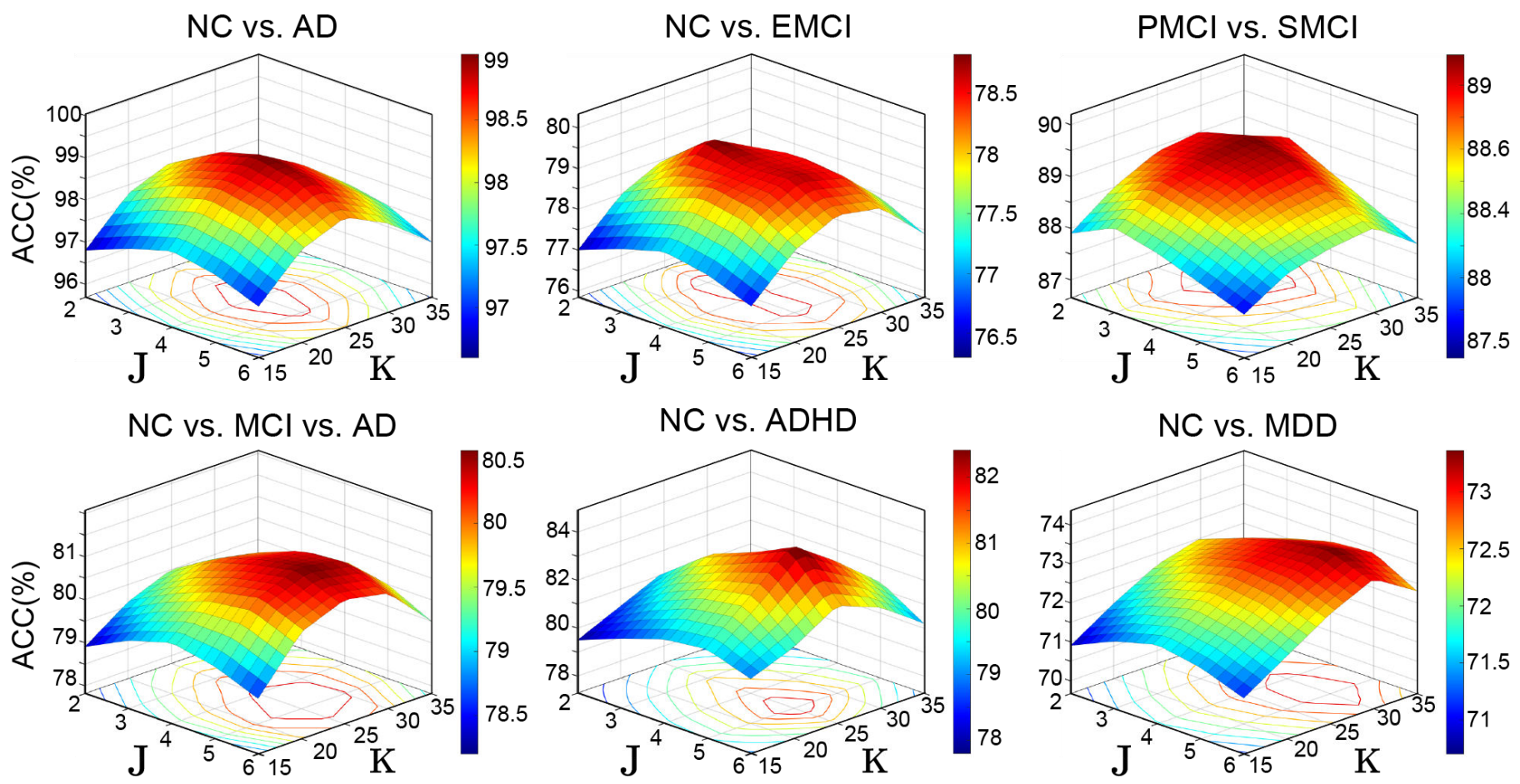

Figure S1: Hyperparameter analysis of J and K for hypergraph construction.

S2.2.2 p-Laplacian orders

We further examined the sensitivity of BriGHT to the p-Laplacian orders used in layer-wise hypergraph regularization. Specifically, we varied p_1 and p_2 for the first and second graph attention layers over a broad range and evaluated performance across all prediction tasks. As shown in Fig. S2, intermediate p values generally perform well, with the best results primarily obtained when p lies in an intermediate range. These results suggest that allowing flexible p-Laplacian smoothing can help balance transcriptomic structural regularization and subject-specific neuroimaging variation.

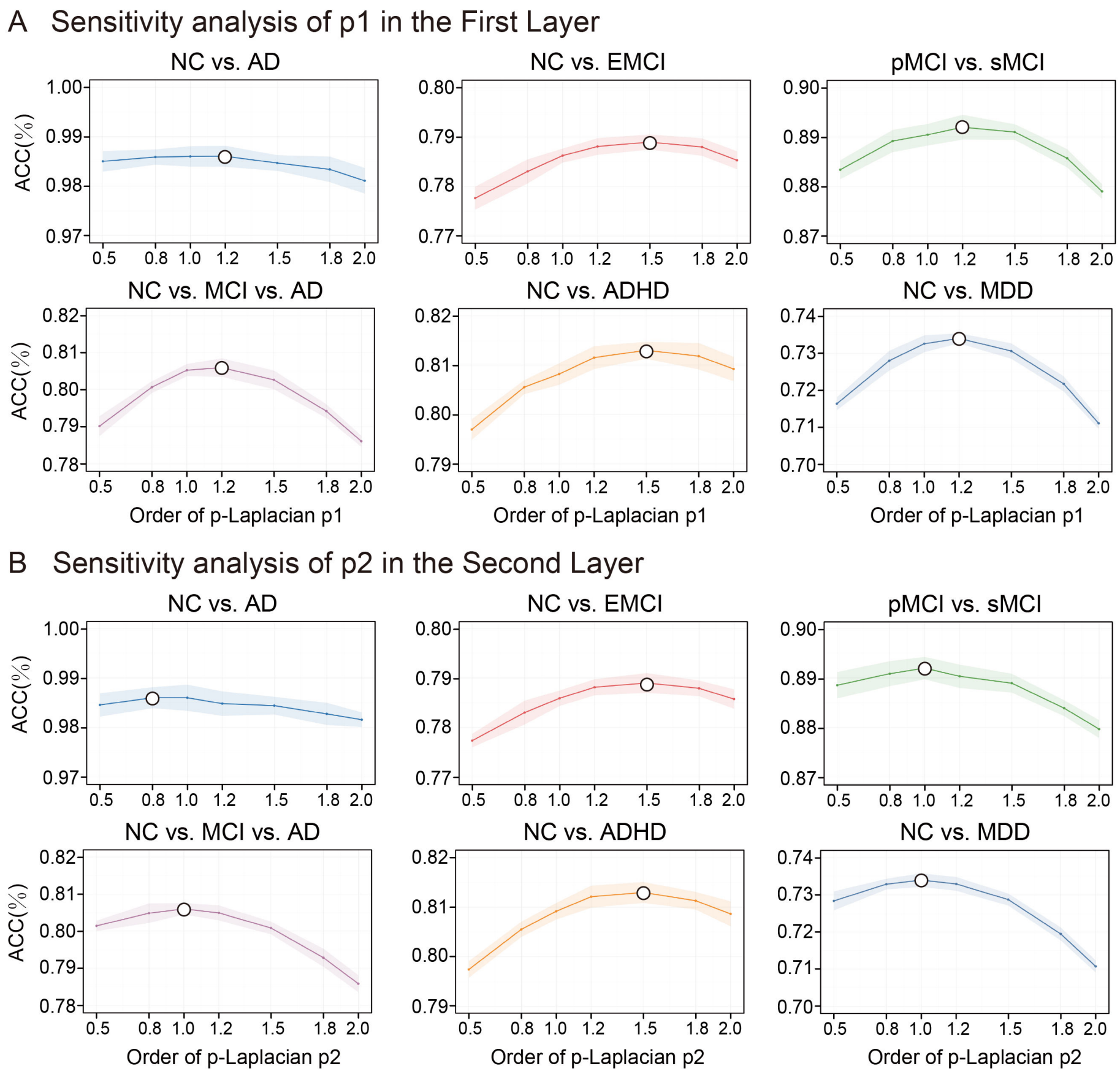

Figure S2: Hyperparameter analysis of the p-Laplacian orders (p_1, p_2). Solid lines denote mean performance across 5-fold cross-validation, and shaded regions indicate standard error. Best configurations are marked with white dots.

S2.3 Sensitivity analysis of transcriptomic regularization and confidence estimation

We evaluated the sensitivity of BriGHT to the transcriptomic regularization weight λ and the confidence-estimation weight η over the candidate set $\{0.5, 0.8, 1.0, 1.2, 1.5, 1.8, 2.0\}$. As shown in Fig. S3, the ACC curves are stable across the evaluated ranges for all six classification tasks, indicating that BriGHT is not sensitive to the hyperparameters.

S2.4 Ablation analysis of reliability component in fusion module

We examine the contribution of each reliability component in the fusion module via a component-wise ablation study. Prediction confidence, cross-modal consistency, and decision certainty are accordingly removed from the fusion module, together with a uniform-fusion baseline. The experiments were conducted on the NC vs. EMCI task. As shown in Table S4, removing any individual component leads to a performance decrease, while the complete fusion strategy achieves the best overall performance, supporting the complementary contributions of the three reliability components.

Table S4: Ablation of reliability components in the fusion module on the NC–EMCI task.

Setting	ACC (%)	AUC (%)
Uniform fusion	77.5 ± 1.3	76.1 ± 1.5
w/o prediction confidence	77.9 ± 1.2	76.5 ± 1.4
w/o cross-modal consistency	78.2 ± 1.1	76.9 ± 1.3
w/o decision certainty	78.5 ± 1.0	77.1 ± 1.3
Full reliability-aware fusion	$\mathbf{78.9 \pm 0.9}$	$\mathbf{77.4 \pm 1.2}$

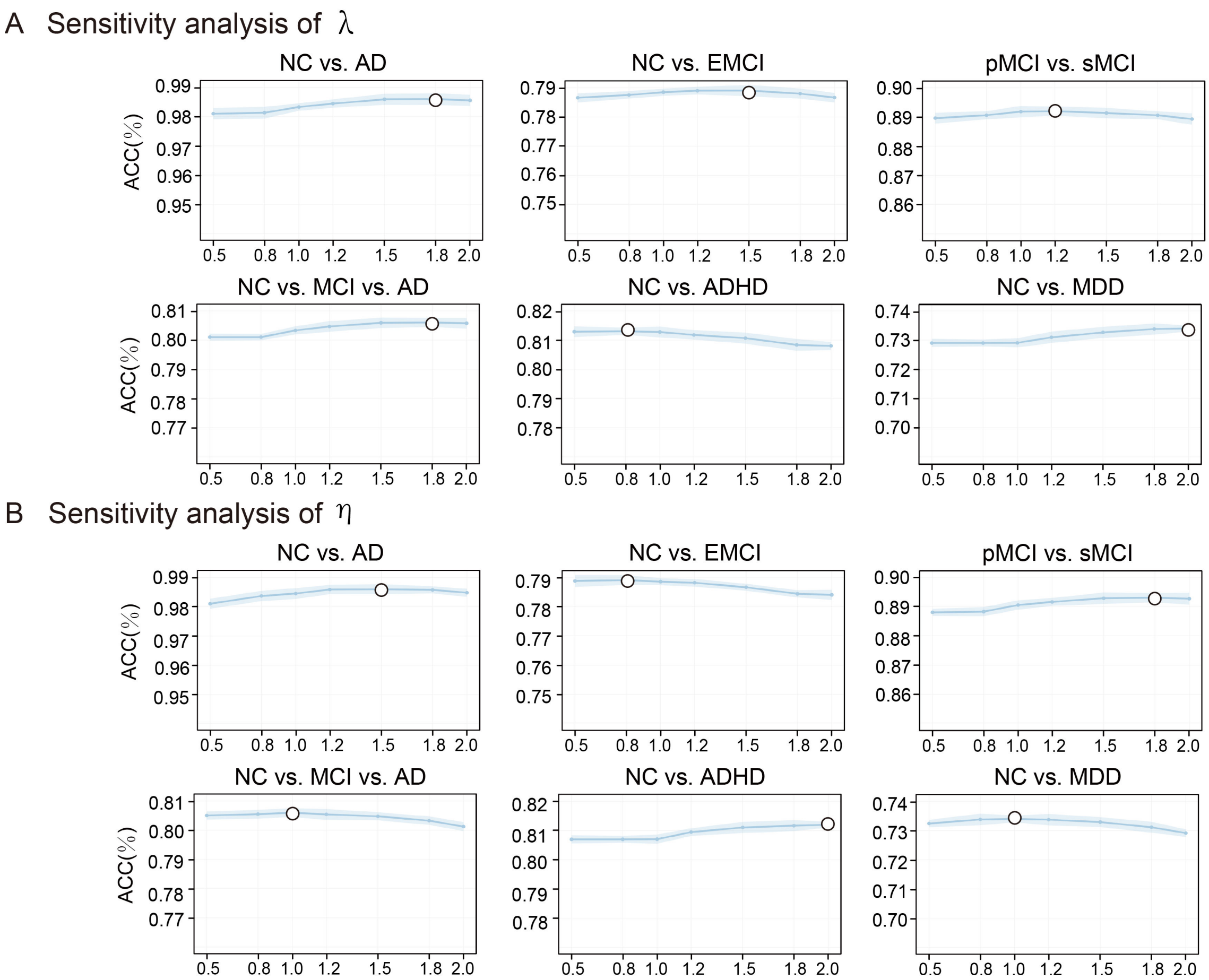

Figure S3: Sensitivity analysis of the hyperparameters involved in Eq. (12). (A) Sensitivity to the transcriptomic regularization weight λ. (B) Sensitivity to the confidence-estimation weight η.

S2.5 Representation robustness analysis

Representation visualization. We visualized representations from three stages: the original imaging features, the representations after transcriptome-regularized graph learning, and the fused multimodal embeddings. As shown in Fig. S4A, diagnostic groups substantially overlap in the original feature space, but become progressively more separable after graph learning and fusion. This suggests that the proposed modules improve the discriminative organization of the latent space.

Robustness analysis. We further evaluated robustness by randomly masking different proportions of input features and comparing the resulting performance degradation. As shown in Fig. S4B, performance decreases as the masking ratio increases, but BriGHT maintains higher accuracy than competing methods across masking levels and tasks. This indicates that the representations learned from our method are less sensitive to feature-level perturbations, consistent with the stabilizing effect of the proposed regularization and fusion modules.

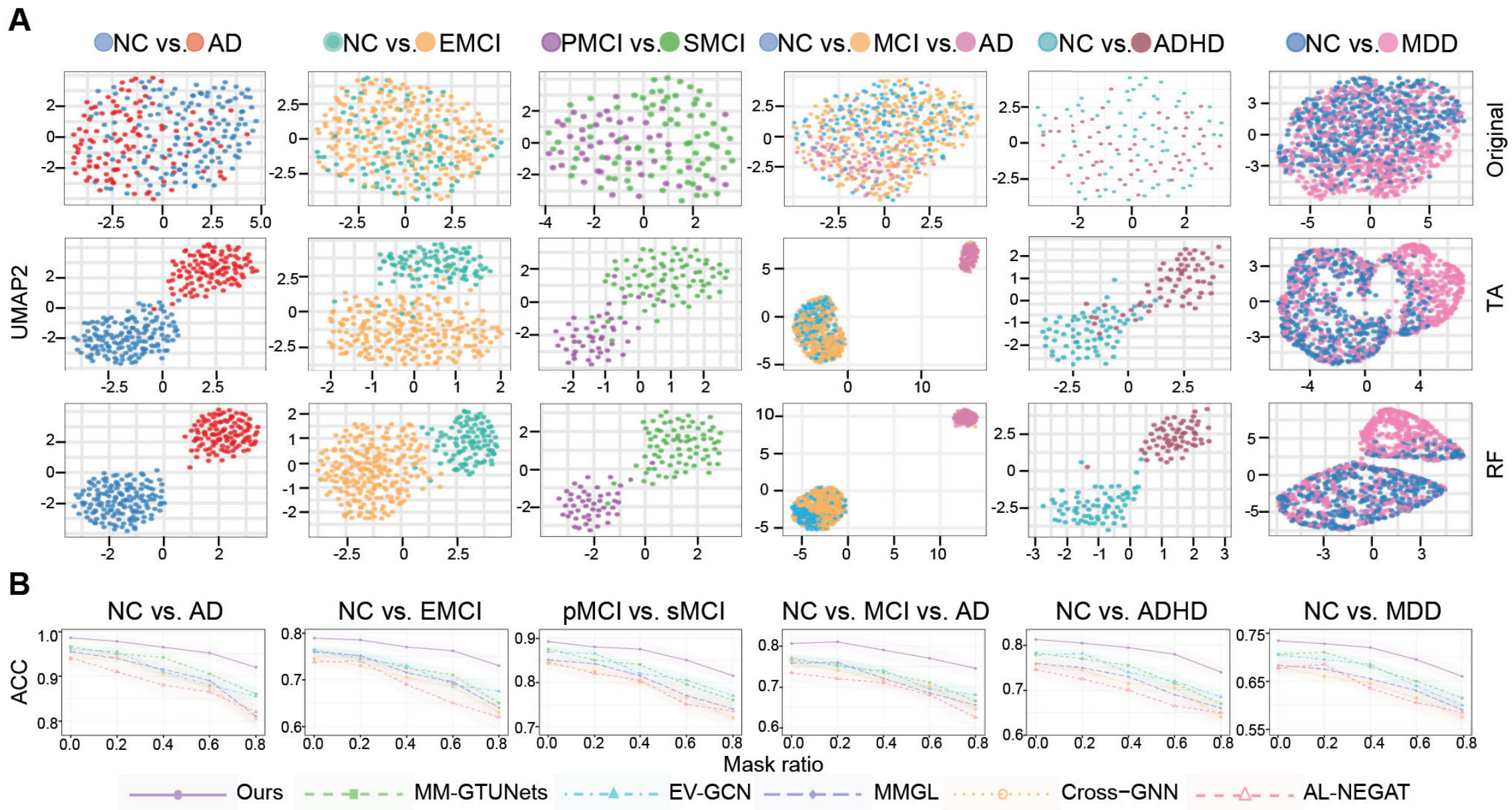

Figure S4: Representation and robustness analysis. (A) UMAP visualization of original features, transcriptome-regularized graph representations, and fused multimodal embeddings. (B) Robustness evaluation under increasing feature masking ratios.

S2.6 Supplementary biological interpretability analysis

Fig. S5 provides additional visualization of the top ROIs identified by feature ablation. Panel A shows the top-10 ROIs for each prediction task, and Panel B presents the corresponding NeuroQuery-derived functional relevance maps. These results complement the main-text interpretability analysis by showing that the identified regions exhibit task-specific spatial patterns and overlap with literature-supported functional relevance maps.

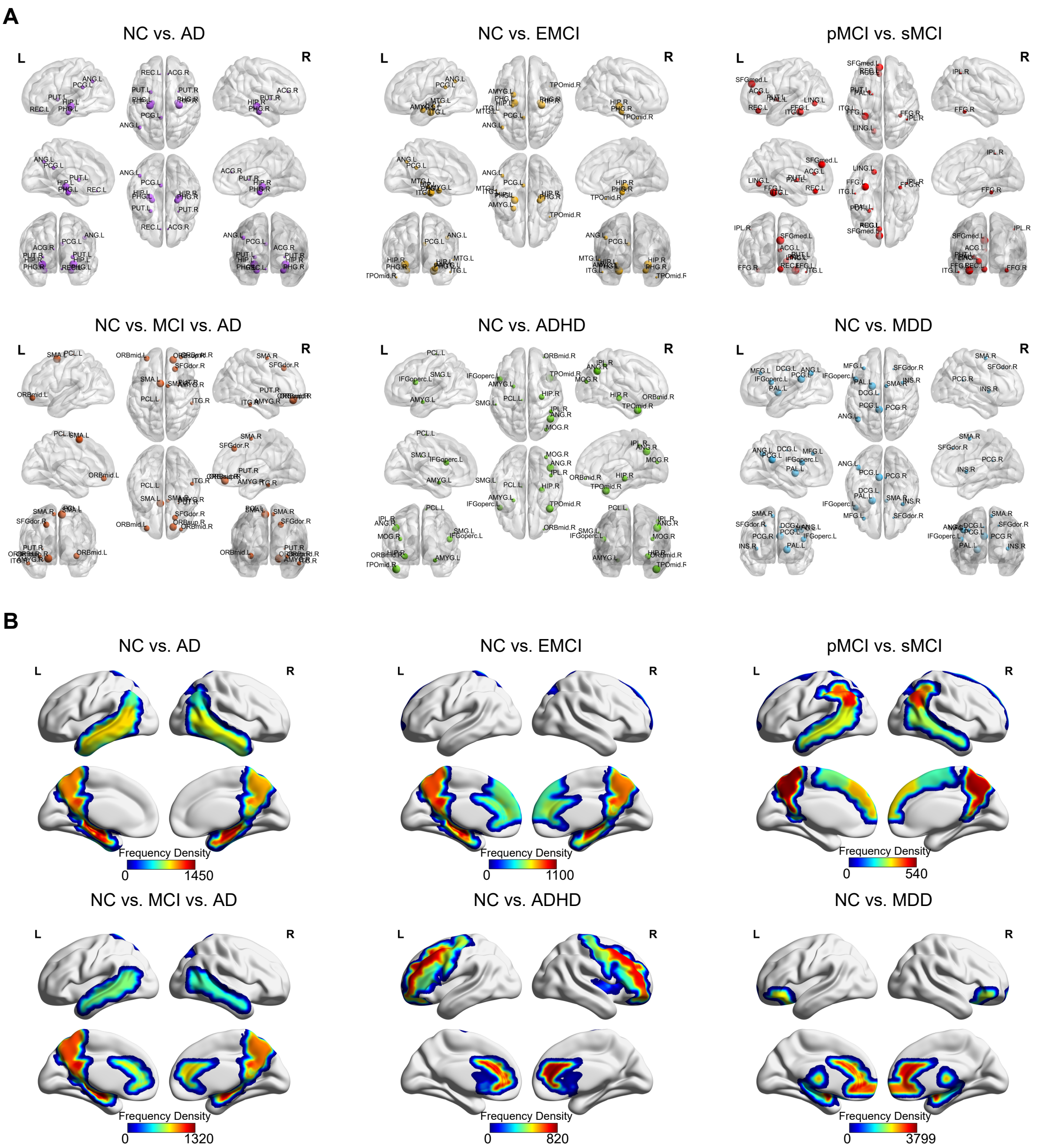

Figure S5: Discriminative brain ROIs and their functional relevance. (A) Top 10 discriminative regions and (B) their literature-derived functional relevance from NeuroQuery meta-analysis.

S2.7 Pathway enrichment analysis of disease-associated genes

We performed GO and KEGG pathway enrichment analyses to assess the biological relevance of the disease-associated genes used for hypergraph construction. As shown in Fig. S6, AD-associated genes were enriched in immune-related signaling, synaptic organization, and hormone-related pathways; ADHD-associated genes were mainly related to neurodevelopmental and synaptic processes; and MDD-associated genes were enriched in synapse-related processes, neuronal signaling, and glutamatergic transmission. These results are broadly consistent with known biological mechanisms of the corresponding disorders and support the use of these gene sets for constructing transcriptome-derived structural references.

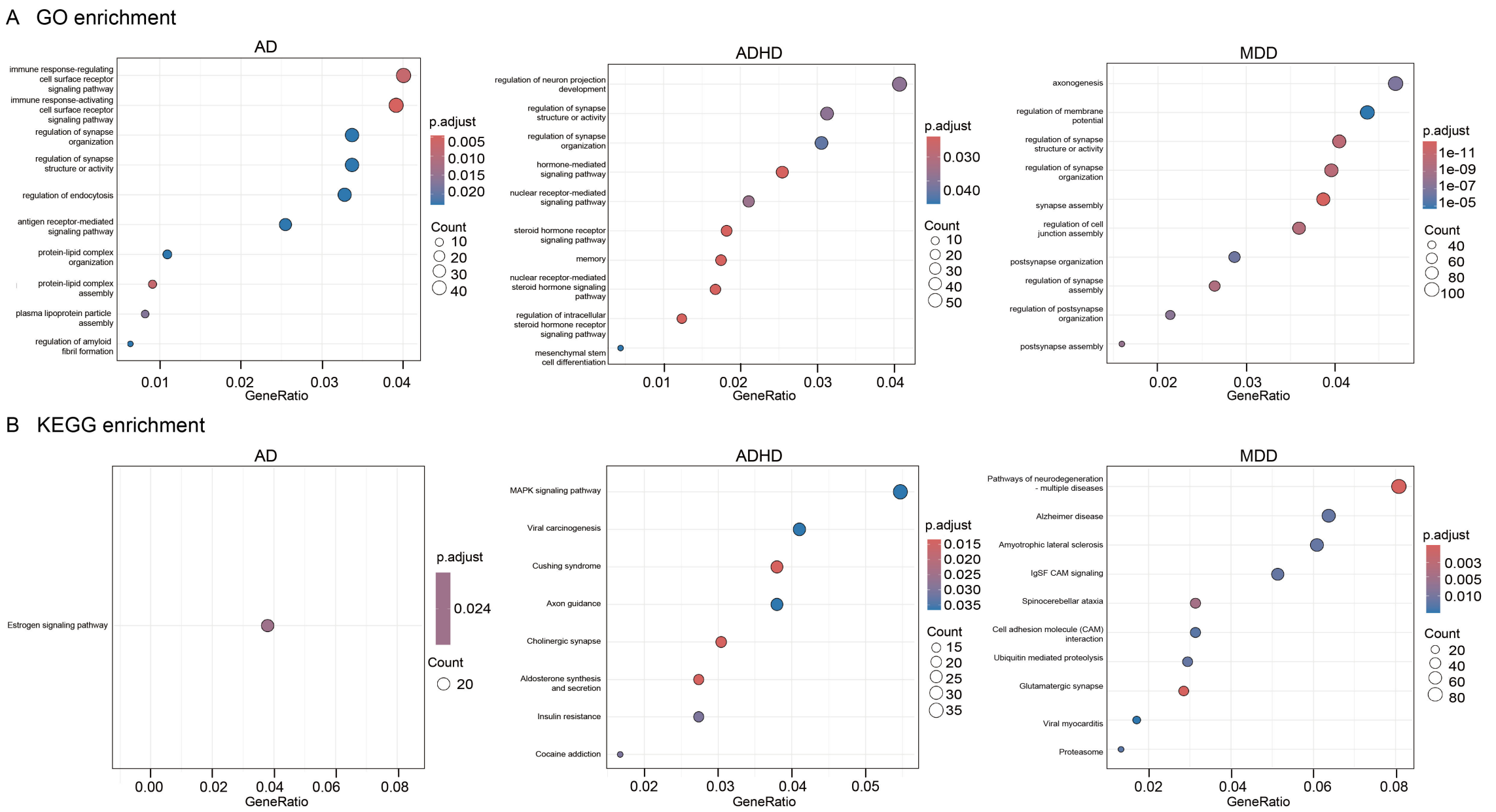

Figure S6: Pathway enrichment analysis of disease-associated genes used for hypergraph construction. (A) GO enrichment results for AD, ADHD, and MDD. (B) KEGG pathway enrichment results for AD, ADHD, and MDD.

Supplemental Reference

[1] RD Markello et al. Standardizing workflows in imaging transcriptomics with the abagen toolbox. *eLife*, 10:e72129, 2021.

[2] Brian W Kunkle et al. Genetic meta-analysis of diagnosed alzheimer's disease identifies new risk loci and implicates aβ, tau, immunity and lipid processing. *Nature genetics*, 51(3):414–430, 2019.

[3] Ditte Demontis et al. Discovery of the first genome-wide significant risk loci for attention deficit/hyperactivity disorder. *Nature genetics*, 51(1):63–75, 2019.

[4] David M Howard et al. Genome-wide meta-analysis of depression identifies 102 independent variants and highlights the importance of the prefrontal brain regions. *Nature neuroscience*, 22(3):343–352, 2019.

[5] Christiaan A de Leeuw et al. Magma: generalized gene-set analysis of gwas data. *PLoS computational biology*, 11(4):e1004219, 2015.

[6] William J Jagust, Dan Bandy, Kewei Chen, Norman L Foster, Susan M Landau, Chester A Mathis, Julie C Price, Eric M Reiman, Daniel Skovronsky, Robert A Koeppe, et al. The alzheimer's disease neuroimaging initiative positron emission tomography core. *Alzheimer's & Dementia*, 6(3):221–229, 2010.

[7] Jingwen Yan, Lei Du, Sungeun Kim, Shannon L Risacher, Heng Huang, Jason H Moore, Andrew J Saykin, Li Shen, and Alzheimer's Disease Neuroimaging Initiative. Transcriptome-guided amyloid imaging genetic analysis via a novel structured sparse learning algorithm. *Bioinformatics*, 30(17):i564–i571, 2014.

[8] Nathalie Tzourio-Mazoyer, Brigitte Landeau, Dimitri Papathanassiou, Fabrice Crivello, Octave Etard, Nicolas Delcroix, Bernard Mazoyer, and Marc Joliot. Automated anatomical labeling of activations in spm using a macroscopic anatomical parcellation of the mni mri single-subject brain. *Neuroimage*, 15(1):273–289, 2002.

[9] Xiaohui Yao, Jingwen Yan, Kefei Liu, Sungeun Kim, Kwangsik Nho, Shannon L Risacher, Casey S Greene, Jason H Moore, Andrew J Saykin, Li Shen, et al. Tissue-specific network-based genome wide study of amygdala imaging phenotypes to identify functional interaction modules. *Bioinformatics*, 33(20):3250–3257, 2017.

[10] Chao-Gan Yan, Xin-Di Wang, Xi-Nian Zuo, and Yu-Feng Zang. Dpabi: data processing & analysis for (resting-state) brain imaging. *Neuroinformatics*, 14:339–351, 2016.

[11] Viktor Sip, Meysam Hashemi, Timo Dickscheid, Katrin Amunts, Spase Petkoski, and Viktor Jirsa. Characterization of regional differences in resting-state fmri with a data-driven network model of brain dynamics. *Science Advances*, 9(11):eabq7547, 2023.

[12] Mi Hyun Lee, Nambeom Kim, Jaeeun Yoo, Hang-Keun Kim, Young-Don Son, Young-Bo Kim, Seong Min Oh, Soohyun Kim, Hayoung Lee, Jeong Eun Jeon, et al. Multitask fmri and machine learning approach improve prediction of differential brain activity pattern in patients with insomnia disorder. *Scientific reports*, 11(1):9402, 2021.
